# CEACAM1 mediates B cell aggregation in central nervous system autoimmunity

**DOI:** 10.1038/srep29847

**Published:** 2016-07-20

**Authors:** Damiano M. Rovituso, Laura Scheffler, Marie Wunsch, Christoph Kleinschnitz, Sebastian Dörck, Jochen Ulzheimer, Antonios Bayas, Lawrence Steinman, Süleyman Ergün, Stefanie Kuerten

**Affiliations:** 1 Department of Anatomy and Cell Biology, University of Würzburg, Würzburg, Germany; 2 Department of Neurology, University Hospitals of Würzburg, Würzburg, Germany; 3 Department of Neurology, Caritas-Krankenhaus Bad Mergentheim, Bad Mergentheim, Germany; 4 Department of Neurology, Klinikum Augsburg, Augsburg, Germany; 5 Department of Neurology, Stanford University School of Medicine, Palo Alto, CA, USA; 6 University Hospital Essen, Department of Neurology, Essen, Germany

## Abstract

B cell aggregates in the central nervous system (CNS) have been associated with rapid disease progression in patients with multiple sclerosis (MS). Here we demonstrate a key role of carcinoembryogenic antigen-related cell adhesion molecule1 (CEACAM1) in B cell aggregate formation in MS patients and a B cell-dependent mouse model of MS. CEACAM1 expression was increased on peripheral blood B cells and CEACAM1^+^ B cells were present in brain infiltrates of MS patients. Administration of the anti-CEACAM1 antibody T84.1 was efficient in blocking aggregation of B cells derived from MS patients. Along these lines, application of the monoclonal anti-CEACAM1 antibody mCC1 was able to inhibit CNS B cell aggregate formation and significantly attenuated established MS-like disease in mice in the absence of any adverse effects. CEACAM1 was co-expressed with the regulator molecule T cell immunoglobulin and mucin domain −3 (TIM-3) on B cells, a novel molecule that has recently been described to induce anergy in T cells. Interestingly, elevated coexpression on B cells coincided with an autoreactive T helper cell phenotype in MS patients. Overall, these data identify CEACAM1 as a clinically highly interesting target in MS pathogenesis and open new therapeutic avenues for the treatment of the disease.

Multiple sclerosis (MS) is thought to be a chronic autoimmune disease of the central nervous system (CNS) and the most common neurological disorder that leads to irreversible deficits and premature retirement in young adults^1^. Recently, B cell aggregates were found in the meninges of patients with secondary progressive MS (SPMS) and associated with more severe clinical disease and cortical histopathology^2^,^3^,^4^. These aggregates showed features reminiscent of B cell follicles in lymphoid tissue, such as B cell proliferation and differentiation into plasma cells, and the presence of a stromal network producing the B cell chemoattractant CXCL13. In autoimmune diseases B cell aggregation is thought to perpetuate inflammation in the target organ through the local generation of pathogenic lymphocytes or autoantibodies^5^. Of major importance for understanding the role of B cell aggregates in immune-mediated CNS inflammation, we have recently developed a B cell-dependent model of MS that recapitulates B cell aggregate formation observed in brain tissue from MS patients^6^,^7^. Active immunization of C57BL/6 (B6) mice with a fusion protein (MP4) consisting of human myelin basic protein (MBP) and the three hydrophilic domains of proteolipid protein (PLP)^8^ reproducibly induced chronic B cell-dependent experimental autoimmune encephalomyelitis (EAE)^9^,^10^. B cell aggregates were present in the CNS of immunized mice from the onset of clinical symptoms and subsequently organized into ectopic lymphoid tissue^7^,^11^.

CEACAM1 is a cell adhesion molecule, which belongs to the immunoglobulin superfamily and mediates cell-cell interaction by homophilic binding. There are eleven variants of CEACAM1, which are processed by alternative splicing^12^. The cytoplasmic domain contains immunoreceptor tyrosine-based inhibitory motifs (ITIMs), which are essentially involved in immunomodulatory signaling pathways of CEACAM1^12^. Along these lines, CEACAM1 has been shown to act as an immunomodulatory co-receptor on T cells^13^. Treatment with anti-CEACAM1 antibody has been reported to attenuate disease activity in T helper (T_H_) cell 1-mediated murine colitis^14^. In addition, clinical disease severity was augmented in a T cell-dependent EAE model after administration of an anti-CEACAM1 antibody^15^. There are only few reports on the role of CEACAM1 in B cell-mediated immunity. CEACAM1 has been demonstrated to be expressed on all B cell subsets and to be involved in activation, survival and differentiation of mature B cells^16^,^17^. CEACAM1 was also shown to promote CD19-induced B cell aggregation^18^.

This is the first study to investigate the role of CEACAM1 in MS. Targeting CEACAM1 by antibody treatment significantly attenuated EAE and was associated with a reduction of B cell aggregates in the CNS. In MS patients the percentage of CEACAM1^+^ B cells was significantly elevated compared to healthy controls. In addition, we found CEACAM1^+^ B cells in brain infiltrates of MS patients. Finally, treatment with anti-CEACAM1 antibody inhibited aggregation of B cells derived from MS patients *in vitro*. Interestingly, the expression of CEACAM1 on B cells coincided with the presence of the regulator molecule T cell immunoglobulin and mucin domain 3 (TIM-3), while the percentage of T_H_ cells expressing both CEACAM1 and TIM-3 was significantly decreased in RRMS patients.

## Results

### Treatment with anti-CEACAM1 antibody significantly ameliorated B cell aggregate formation in the CNS

**Figure 1:**
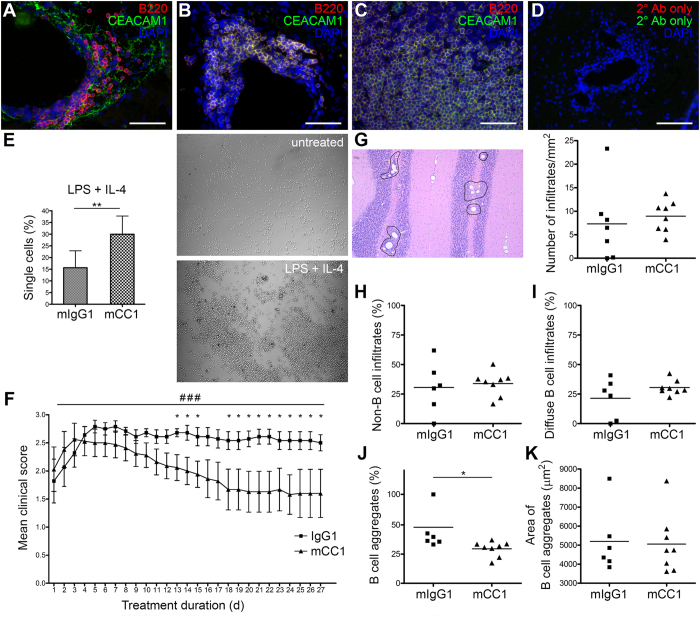
Amelioration of MP4-induced EAE after treatment with mCC1. (**A,B**) CEACAM1 expression on B cells in the CNS in acute (**A**) and chronic (**B**) MP4-induced EAE. (**C**) CEACAM1 expression on B cells in lymph nodes of MP4-immunized mice. (**D**) Negative control staining in the absence of primary antibody. Scale bars: 50 μm. (**E**) Inhibition of murine B cell aggregation induced by 25 μg/ml LPS + 5 U/ml IL-4 after treatment with 200 μg/ml anti-CEACAM1 antibody (mCC1). For each experiment *n* = 3 B6 mice were pooled. B cell aggregation was induced over a period of 72 h (compare images of untreated and LPS + IL-4-stimulated B cells). Data are representative of four independent experiments with similar results. Statistical significance was determined by Wilcoxon rank sum test. Data are expressed as mean ± s.d. *P < 0.05; **P < 0.01; ***P < 0.001. (**F**) Clinical course of EAE after treatment of MP4-immunized B6 mice with mCC1 (*n* = 8) or mIgG1 isotype control antibody (*n* = 7). Treatment was started with disease onset and given every three days. Results are from three independent experiments. Statistical significance was determined by one-way ANOVA for day-to-day comparisons (*P < 0.05) or by Wilcoxon rank sum test for the entire time course (^###^*P* < 0.001). Data are expressed as mean ± s.d. (**G**) Representative image of perivascular infiltrates in the cerebellum of MP4-immunized mice (left panel, circles) and their number after treatment with mCC1 *vs*. mIgG (right panel). (**H–K**) Percentage of non-B cell infiltrates (**H**), diffuse B cell infiltrates (**I**) and B cell aggregates (**J**) and size of B cell aggregates (**K**) in the cerebellum of MP4-immunized mice after treatment with mCC1 *vs*. mIgG1. Statistical significance was determined by Wilcoxon rank sum test. *P < 0.05.

We immunized B6 mice with MP4 and screened CNS infiltrates for the presence of CEACAM1 on B cells both in acute and chronic EAE. Acute EAE was defined as the initial peak of clinically evident EAE, while chronic EAE was assessed 60 days after EAE onset. In acute EAE B cells were disseminated within the CNS infiltrates and did not show any expression of CEACAM1 on the cell surface (Fig. 1A). In chronic EAE typical B cell aggregates were observed as we have described before^7^. In these B cell aggregates almost all B cells were positive for CEACAM1 (Fig. 1B) similar to germinal center B cells within lymph nodes (Fig. 1C). Sections exposed to secondary antibody only were negative for CEACAM1 (Fig. 1D). In a next step, we tested the anti-CEACAM1 antibody clone mCC1 for its anti-aggregative capacity in murine *in vitro* B cell aggregation assays. Purified splenic B cells were stimulated with LPS + IL-4 for 72 h in the presence of 200 μg/ml mCC1 or mIgG1 isotype control antibody. There was a significant increase in the number of single cells when aggregation assays were performed in the presence of mCC1 compared to preincubation with isotype control antibody (Fig. 1E). To determine whether mCC1 also displayed an inhibitory effect on *in vivo* B cell aggregate formation, B6 mice were immunized with MP4 to induce B cell-dependent EAE and B cell aggregate formation in the CNS. Mice developed EAE on day 22.8 ± 5.2 after immunization. Treatment with either mCC1 (*n* = 8) or mIgG1 isotype control antibody (*n* = 7) was initiated at the onset of the disease. The mean EAE score at the time of disease onset was comparable in both groups (2.03 ± 1.12 in mCC1- compared to 1.82 ± 1.03 in mIgG1-treated mice). Mice received intraperitoneal injections every third day for a total period of 27.6 ± 5.4 days after EAE onset. There was a significant reduction in the clinical EAE score in the mCC1-treated group beginning on day 13 after treatment onset (Fig. 1F). To evaluate whether the amelioration of clinical EAE was associated with a reduction of B cell aggregate formation in the CNS the cerebellum was assessed for inflammatory infiltrates at the end of the treatment period. There was no difference in the total number of perivascular infiltrates in the cerebellar parenchyma between the two groups (Fig. 1G, right panel). However, when we classified the infiltrates into non-B cell infiltrates, diffuse B cell infiltrates and B cell aggregates (Fig. 1H–J, Supplementary Fig. 1) we found a significant decrease in B cell aggregate formation in the mCC1-treated group (Fig. 1J). Remarkably, there was no difference in the B cell aggregate size comparing mCC1- and mIgG1-treated mice (Fig. 1K) suggesting that mCC1 mainly targeted the formation of new B cell aggregates while already existing aggregates remained unaffected. We also assessed CEACAM1 expression by B220/mCC1 double staining of *n* = 8 mCC1- and *n* = 6 mIgG1-treated MP4-immunized mice (Supplementary Fig. 2A–C). In mIgG1-treated mice 80.7% ± 15.9% of all infiltrates contained CEACAM1^+^ cells, while this number increased to 94.0% ± 3.8% in B cell aggregates (Supplementary Fig. 2D,E). In mCC1-treated mice there was a strong trend towards a decrease in the percentage of CEACAM1^+^ B cell aggregates (84.4% ± 14.3%), while the percentage of overall CEACAM1^+^ infiltrates remained nearly unchanged (74.6% ± 12.5%) (Supplementary Fig. 2D,E). Besides this decrease in CEACAM1^+^ cells in mice treated with mCC1 antibody, we also noted diminished fluorescence intensity of the mCC1 staining (Supplementary Fig. 2C).

### Anti-CEACAM1 antibody treatment did not trigger any adverse immunological effects

**Figure 2:**
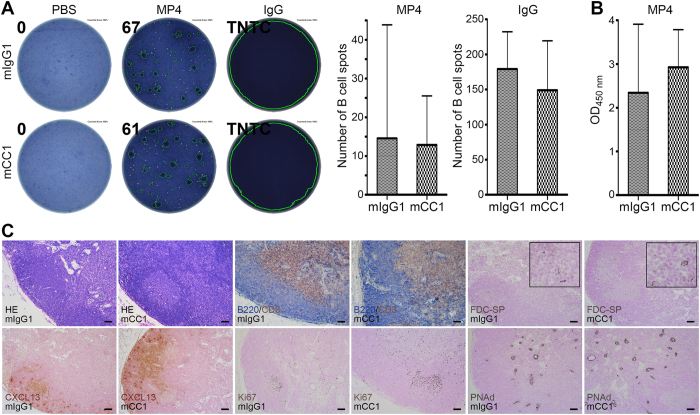
Antigen-specific B cell response and lymph node architecture are unaltered in mice after treatment with mCC1 antibody. (**A**) B cell ELISPOT assay of draining inguinal lymph nodes for the detection of MP4-specific antibodies and total IgG in MP4-immunized mice treated with either mCC1 (*n* = 8) or mIgG1 (*n* = 7). (**B**) Serum ELISA for the detection of MP4-specific antibodies in MP4-immunized mice treated with either mCC1 (*n* = 8) or mIgG1 (*n* = 7). Statistical significance was determined by Wilcoxon rank sum test. (**C**) Representative images of IHC staining of inguinal draining lymph nodes from MP4-immunized mice treated either with mCC1 (*n* = 8) or mIgG1 (*n* = 7). Scale bars: 50 μm.

None of the mice treated with mCC1 showed any clinically evident adverse treatment effects. To confirm that the main mode of action of mCC1 treatment was its anti-aggregative effect on newly developing B cell aggregates in the CNS we also performed B cell ELISPOT on the draining inguinal lymph nodes of MP4-immunized mice (Fig. 2A, left). The data demonstrate that the number of MP4-specific B cells was comparable in mCC1- and mIgG1-treated mice (Fig. 2A, left graph). In addition, there was no significant difference between the amounts of total IgG production by lymph node B cells (Fig. 2A, right graph). Along these lines, there was also no difference in the levels of MP4-specific IgG antibodies as detected in the sera of MP4-immunized mice by ELISA (Fig. 2B). We also analyzed the overall lymph node architecture performing immunohistochemistry (IHC) for typical markers of lymphoid tissue. These included staining for B cell and T cell compartments, CD35^+^ follicular dendritic cells (FDCs), CXCL13, proliferation and peripheral node addressin (PNAd)^+^ high endothelial venules (HEVs) (Fig. 2C). We did not observe any alterations in lymph node architecture after treatment with mCC1. Finally, we isolated splenic B cells from naïve B6 mice and incubated these cells either with 200 μg/ml mCC1 or mIgG1 isotype control antibody. Flow cytometric analysis showed no difference in B cell activation marker expression comparing mCC1 to mIgG1 treatment (Supplementary Fig. 3). Similar results were obtained when cells were stimulated with LPS + IL-4 for 72 h prior to or after incubation with either mCC1 or mIgG1 (data not shown). Taken together, these results support the notion that the observed treatment effect of mCC1 was target-specific and mainly pertained to the inhibition of B cell aggregate formation.

### The percentage of CEACAM1^+^ B cells was increased in patients with RRMS

**Figure 3:**
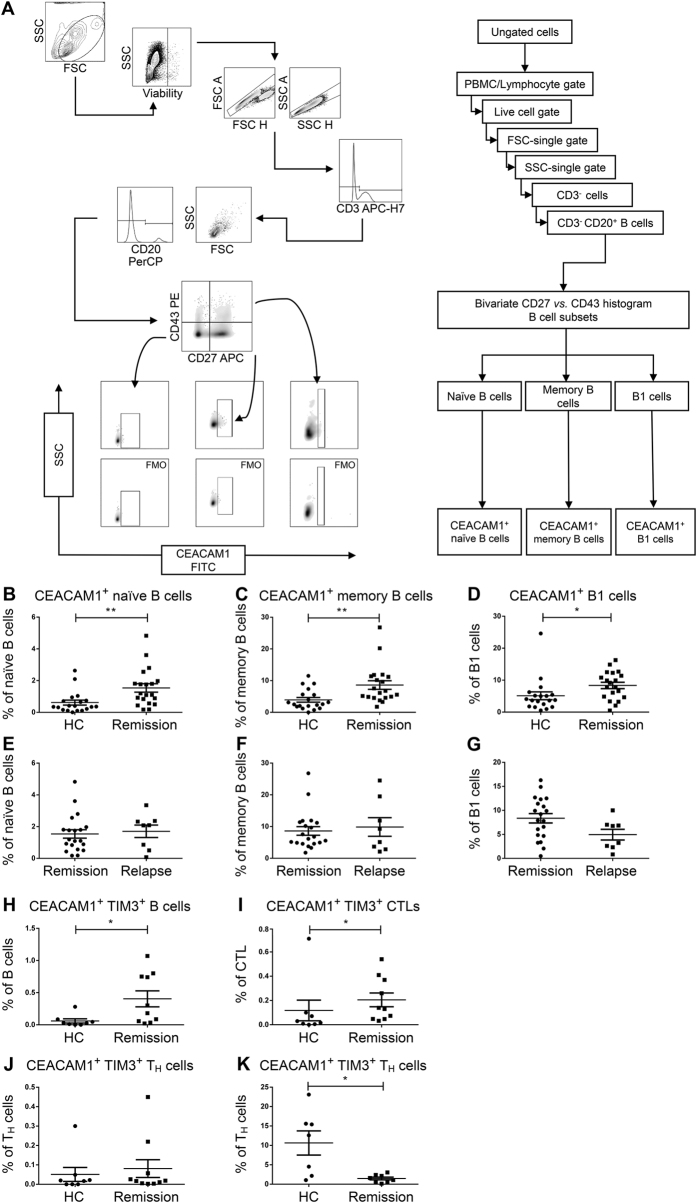
Flow cytometric analysis of CEACAM1 expression on peripheral B cells from healthy controls and RRMS patients. (**A**) Gating strategy. (**B–G**) CEACAM1 expression on naïve (**B,E**) and memory B cells (**C,F**) as well as on B1 cells (**D,G**). MS patients were tested during remission (*n* = 19) (**B–D**) and relapse (*n* = 8) (**E–G**). Statistical significance was determined by Wilcoxon rank sum test. (**H–K**) CEACAM1/TIM-3 coexpression on B cells (**H**), cytotoxic T lymphocytes (CTLs, **I**) and T helper cells (T_H_, **J,K**) before (**J**) and after (**K**) anti-CD3/anti-CD28 antibody stimulation. Samples were analyzed from patients during remission that received treatment with natalizumab (before stimulation *n* = 10, after stimulation *n* = 8). Healthy controls (*n* = 8*, n* = 7) were age- and gender-matched. Patients in remission all received natalizumab (*n* = 19), while patients in relapse received either natalizumab (*n* = 1), interferon-β (*n* = 2) or were untreated (*n* = 5).

In an attempt to translate our findings from the animal model to the setting of MS we obtained venous blood samples from patients with RRMS both during remission and relapse and from age- and gender-matched healthy controls (Supplementary Table 1). Peripheral blood mononuclear cells (PBMCs) were stained for the expression of CEACAM1 on naïve and memory B cells as well as on the B1 cell subset and the samples were analyzed by polychromatic flow cytometry (Fig. 3A). The data suggest that RRMS patients displayed a significant increase in the percentages of CEACAM1^+^ cells that pertained to all of the three B cell subsets that were analyzed (Fig. 3B–D). Interestingly, there was no difference in the percentages of CEACAM1^+^ B cell subsets comparing remission and relapse (Fig. 3E–G). In a next step, we polyclonally stimulated PBMCs with the TLR7/8 agonist R-848 and IL-2. In this setting, the percentage of CEACAM1^+^ B cells was also increased in RRMS patients compared to healthy controls (Supplementary Fig. 4, Supplementary Tables 2 and 3). Performing subset analysis we noted that this increase mainly pertained to the B1 cell subset (Supplementary Fig. 4D). Our analysis of CEACAM1 expression on both unstimulated and stimulated T_H_ and CD8^+^ cytotoxic T cells (CTLs) revealed that there was no difference between RRMS patients and healthy controls (Supplementary Fig. 5, Supplementary Tables 2 and 3). Recently published data suggest an interaction of CEACAM1 with TIM-3 on T cells as a key mechanism for induction^13^. We assumed that a similar mechanism could also apply to B cells. To determine whether B cells actually express TIM-3 we first performed PCR and immunocytochemistry (ICC) on enriched human B cells compared to PBMCs. In all individuals that were analyzed (both healthy controls (PCR and ICC) and RRMS patients (PCR)) TIM-3 was expressed in B cells (Supplementary Fig. 6) and on the surface of a small number of B cells (Supplementary Fig. 7). To elucidate a potential interaction between CEACAM1 and TIM-3 we next stained PBMCs from RRMS patients for the expression of both CEACAM1 and TIM-3 (Fig. 3H–K, Supplementary Table 4) and observed a significant increase in the fraction of CEACAM1^+^TIM-3^+^ B cells and CTLs in RRMS patients (Fig. 3H,I). Since we did not observe TIM-3 expression on unstimulated T_H_ cells (Fig. 3J), we treated PBMCs with anti-CD3/anti-CD28 antibodies for 72 h, which induced TIM-3 expression on T_H_ cells (data not shown). Interestingly, the percentage of CEACAM1^+^TIM3^+^ T_H_ cells was significantly decreased in RRMS patients (Fig. 3K).

### CEACAM1^+^ B cells were present in brain infiltrates of MS patients

**Figure 4:**
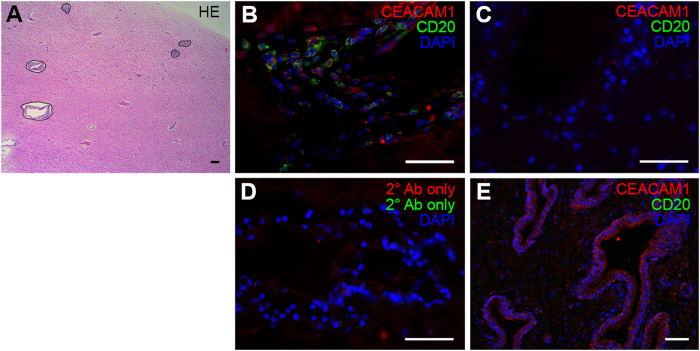
CEACAM1^+^ B cells are present within brain infiltrates of MS patients. (**A**) Overview of brain infiltration in MS showing perivascular infiltrates stained by H.E. (**B**) IHC staining of CEACAM1 in MS brain infiltrates that contain B cell infiltration. (**C**) IHC staining of non-MS control brain tissue. (**D**) IHC control in the absence of primary antibody. (**E**) Positive control staining of human prostate tissue. Data are representative of *n* = 12 MS and *n* = 12 non-MS cases. Scale bars: 50 μm.

To collect evidence for the presence of CEACAM1^+^ B cells in the CNS of MS patients, we used *post mortem* brain sections from *n* = 12 MS patients (Supplementary Table 5), which contained lymphocytic infiltration (Fig. 4A) for IHC. Brain samples from *n* = 12 healthy donors served as a control. There were two patient cases displaying B cell aggregates that resembled the ones that were found in the meninges of MS patients^3^,^5^. In both cases CEACAM1 was expressed on the surface of the aggregated B cells. Diffuse B cell infiltrates were found in all cases. CEACAM1^+^CD20^+^ cells within these diffuse infiltrates were found in *n* = 4 cases. While 52.44 ± 6.32% of B cells were CEACAM1^+^ in B cell aggregates, 43.25 ± 32.63% of B cells expressed CEACAM1 in diffuse infiltrates (Fig. 4B). None of the controls displayed any lymphocytic infiltration (Fig. 4C). Control sections exposed to secondary antibody only were negative (Fig. 4D), while human prostate sections served as a positive control displaying epithelial CEACAM1 expression (Fig. 4E).

### Anti-human CEACAM1 antibody was equally effective in inhibiting *in vitro* B cell aggregation in both healthy controls and RRMS patients

**Figure 5:**
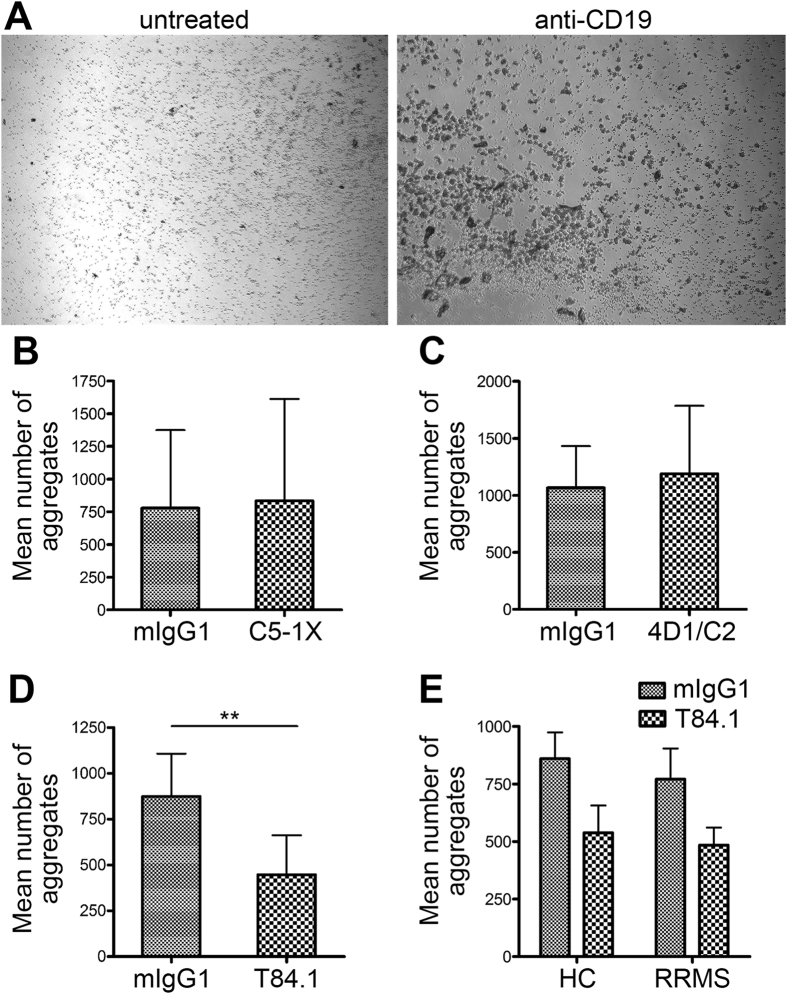
Inhibition of *in vitro* B cell aggregation in MS patients. B cell aggregation was induced by 16 h of incubation with anti-CD19 (**A**, compare untreated to anti-CD19-treated cells). (**B**–**D**) Inhibition of CD19-induced B cell aggregation by the anti-CEACAM1 antibody clones C5-1X (150 μg/ml) (**B**), 4D1/C2 (150 μg/ml) (**C**) and T84.1 (150, 250 *vs*. 400 μg/ml) (**D**). B cells were isolated from *n* = 3 (**B,C**) or *n* = 9 (**D**) healthy donors, respectively. (**E**) Induction of B cell aggreation by anti-CD19 antibody and its inhibition by T84.1 in age- and gender-matched MS patients (*n* = 4) and healthy controls (*n* = 4).

In order to assess whether the aggregation of B cells derived from MS patients could be inhibited by using anti-CEACAM1 antibody we used the human *in vitro* B cell aggregation assay as initially described by Lobo *et al*.^18^ (Fig. 5A, both panels). In a first step, we tested three different clones for their anti-aggregative capacity using B cells from healthy controls. While the clone C5-1X targets the A1-B-domain of CEACAM1, 4D1/C2 is directed against the linker between the B1-A2 domain and T84.1 recognizes the N-terminus of the molecule. The data demonstrate that both C5-1X (Fig. 5B) and 4D1/C2 (Fig. 5C) were not capable of inhibiting B cell aggregation in healthy controls. In contrast, preincubation with T84.1 significantly reduced subsequent B cell aggregation induced by anti-CD19 (Fig. 5D). We tested three different concentrations of T84.1 and did not find any difference between 150 μg/ml, 250 μg/ml and 400 μg/ml. Therefore, data for these three concentrations were pooled in Fig. 5D. For the subsequent comparison between MS patients and age- and gender-matched healthy controls (Supplementary Table 7) we used a concentration of 400 μg/ml of T84.1. As shown in Fig. 5E both the induction of B cell aggreation by anti-CD19 antibody and its inhibition by T84.1 were comparable between healthy controls and MS patients.

## Discussion

The present study provides several lines of evidence that identify CEACAM1 as a key molecule in B cell-dependent EAE and MS immunopathology. Briefly, treatment with CEACAM1-specific antibody resulted in a reduction of B cell aggregate formation both *in vitro* and *in vivo* as well as in clinically attenuated B cell-dependent EAE. Furthermore, CEACAM1 expression was elevated on all major human B cell subsets and CEACAM1^+^ B cells were found in brain infiltrates of MS patients. Finally, our data suggest that CEACAM1 and TIM-3 act as immune checkpoint molecules on autoreactive B and T cells.

Different molecules have been proposed to play a key role in the formation of ectopic lymphoid tissue including lymphotoxin (LT) and the homeostatic chemokines CXCL13, CCL19 and CCL21^19^. While CEACAM1 expression on B cells was reported earlier^17^, the first report showing a supportive role of CEACAM1 in the formation of B cell aggregates was published by Lobo *et al*.^18^. The novel aspect of our study was that we did not only observe CEACAM1 expression on B cell aggregates within the lymph node, but also on B cells within the CNS itself. In MP4-induced EAE CEACAM1 expression on B cells did not occur at the stage of diffuse B cell infiltration, however, it was a characteristic feature of B cell aggregates in chronic EAE. CEACAM1^+^ B cells were also present in brain infiltrates of progressive MS patients. This result was further supported by flow cytometric analysis demonstrating that CEACAM1 was expressed on all major B cell subsets, while the percentage of B cells expressing CEACAM1 was significantly increased in the blood of MS patients compared to healthy controls. We observed no differences in the number of CEACAM1^+^ B cells comparing relapse and remission patients. However, it has to be noted that patients in remission were on natalizumab treatment, while relapse patients received different therapies. Therefore, future studies will clearly have to deal with treatment effects on CEACAM1^+^ B cells. Recent evidence indicates that the expression of CEACAM1 and TIM-3 marks a population of exhausted T cells^13^. We observed a significant decrease in CEACAM1^+^TIM-3^+^ T_H_ cells after T cell stimulation in RRMS patients, which is likely to be reflective of the autoimmune nature of the disease. These data are in line with previously published data by others that demonstrated a loss of TIM-3 regulation of T cell function in untreated MS patients^20^. Interestingly, RRMS patients displayed increased frequencies of CEACAM1^+^TIM-3^+^ B cells and CTLs compared to healthy controls. To our knowledge, this is the first report to demonstrate the expression of TIM-3 on B cells. In addition, we suggest that the subset of CEACAM1^+^TIM-3^+^ B cells may play a key yet to be explored role in the pathogenesis of the disease. On the one hand, the increased frequencies of CEACAM1^+^TIM-3^+^ B cells and CTLs may be a direct result of the autoimmune processes in MS reflecting the physiological mechanisms of induction in T_H_ cells^13^, which may also be applied to B cells and CTLs. Accordingly, MBP-stimulated TIM-3 expressing CD8^+^ T cells were significantly augmented in RRMS patients compared to healthy controls^21^. In addition, the observed frequencies of CEACAM1^+^TIM-3^+^ B cells and CTLs were in the order of 1% resembling the low frequencies of autoreactive IgM^+^ memory B cells and T lymphocytes in the peripheral blood^22^,^23^. Polyclonal B cell stimulation was able to induce CEACAM1 expression on T_H_ cells, which was therefore not defective *per sē* in RRMS patients. However, we observed a significant reduction of CEACAM1^+^ T_H_ cells in RRMS patients after T cell stimulation with anti-CD3/anti-CD28 compared to healthy controls suggesting that B cells are crucially involved in inducing CEACAM1 on T_H_ cells (data not shown). While the aim of this study was not to investigate the mechanisms behind the decrease in CEACAM1^+^TIM-3^+^ T_H_ cells in RRMS we suggest that the increased CEACAM1^+^ B cell frequencies were a direct consequence of this defect. In addition to what has been described by Huang *et al*.^13^ CEACAM1 may not only recruit TIM-3 by *cis* binding on the same cells, but also on a different cell in *trans* manner. Along these lines increased frequencies of CEACAM1^+^ B cells in RRMS patients may result from their futile attempt to induce anergy via the CEACAM1/TIM-3 axis on defective T_H_ cells. In this scenario, B cell aggregation occurs as a side effect of these aforementioned aberrant immune processes in B cell-driven MS.

The presence of CEACAM1 on B cell aggregates prompted us to determine whether B cell aggregate formation in the CNS could be therapeutically targeted using anti-CEACAM1 antibodies. Since the clone mCC1 demonstrated an anti-aggregative effect in our murine *in vitro* B cell aggregation pre-testing experiments, we selected this clone for injection into mice. The injection of mCC1 led to a significant reduction in the clinical disease scores even when administered after disease onset. The rationale behind a treatment initiation after onset of EAE was that we wanted to focus on the relationship between CEACAM1 and B cell aggregate formation while avoiding any effects on the development of the autoreactive T cell response, which determines the inductive phase of the disease^24^,^25^. Overall, our results were remarkable because the increasing reduction in clinical disease severity over time during mCC1 treatment coincided with the kinetics of B cell aggregate formation in the CNS which we have previsouly extensively characterized in this model^7^,^11^. Indeed, while there was no difference in the percentage of both non-B cell and diffuse B cell infiltrates comparing mCC1- and isotype control-treated mice the percentage of B cell aggregates was significantly reduced after mCC1 treatment. Of note, there was no difference in the B cell aggregate size, which suggests that mCC1 treatment was specifically effective in preventing the formation of new B cell aggregates, while already existing aggregates at the timepoint of treatment remained unaffected.

CEACAM1 is a molecule that is expressed on different cells types including angiogenically activated endothelial cells, leukocytes and epithelia^12^. Basically, the adhesive and morphogenetic function of CEACAM1 is independent of the cell type as it has been shown for new vessels^26^,^27^ and epithelial lumen formation^28^. It is reasonable to assume that the adhesive function of CEACAM1 is the driving mechanism behind the formation of B cell aggregates in the CNS. However, due to the functional heterogeneity of the molecule we reasoned that treatment with anti-CEACAM1 could also induce side effects outside CNS B cell aggregates, in particular on already exisiting B cell follicles in secondary lymphoid organs. At this stage our data suggest that our treatment neither affected basal B cell functions nor the morphology of B cell areas within lymph nodes. Yet, CEACAM1 has recently been proposed to promote the survival of activated mature B cells^16^. In addition, CEACAM1 has been shown to function as an inihibitory co-receptor on T cells and the same is assumed for B cells^13^,^17^,^18^. To this end, we cannot exclude additional effects of anti-CEACAM1 treatment on B cell differentiation and function *in situ*. Since in particular the clone mCC1 has been described to function as an activating antibody on CEACAM1^16^, it is conceivable that the observed reduction in EAE severity after treatment could at least in part be assigned to an additional inhibition of the B cell-mediated immune response including a reduction in local autoantibody production. Still, we believe that if there is indeed such an additional treatment effect, it might be rather marginal in its nature since we did not observe any reduction in MP4- or total IgG production in the draining lymph nodes. It also has to be noted that all mice survived the treatment and did not show any clinical signs that might have been related to adverse effects of mCC1 application. The observation period of about 30 days, however, does not allow to draw any definite conclusion as to safety and potential side effects and data on the use of this antibody in humans are lacking. Future studies will also have to deal with delineating additional effects of anti-CEACAM1 treatment on both the autoreactive B and T cell response in EAE and MS. At this point we cannot exclude any negative effects on the recruitment of immune checkpoint molecules such as TIM-3. We will also have to consider the effect of CEACAM1 on innate immune cells before translating our concept as put forward in the current study into clinical trails. Taken together, our data are promising for the establishment of new and more specific therapeutic strategies in MS that target CEACAM1 and thereby reduce the detrimental effects of B cell-mediated autoimmunity in the CNS.

## Methods

### Mice

Female six-week-old wild-type B6 mice were purchased from Janvier and maintained under specific pathogen-free conditions in the animal facilities of the Department of Dental Medicine at the University of Würzburg. Mice were maintained in individually ventilated cages with autoclaved woodchip bedding in groups of two to six mice. Mice were fed a standard rodent diet (Altromin) and had free access to pathogen-free water. From the time when mice displayed paralytic signs feed and water were offered at ground level. All animal experiments complied with the German Law on the Protection of Animals, the ‘Principles of laboratory animal care’ (NIH publication No. 86–23, revised 1985) and the ARRIVE guidelines. The treatments were performed according to a protocol that was approved by the Regierung von Unterfranken, Germany (approval number 114/13).

### EAE induction

Mice were injected with a total dose of 200 μg MP4 (Alexion Pharmaceuticals) emulsified in an equal volume of complete Freund’s adjuvant (CFA) by subcutaneous application (total injection volume: 200 μl). CFA contained a 1:9 mixture of mannide monooleate (Sigma) and paraffin oil (Roth) and 5 mg/ml of Mycobacterium tuberculosis H37 Ra (Difco Laboratories). Pertussis toxin (List Biological Laboratories) was given intraperitoneally at 200 ng/mouse on the day of immunization and 48 h later. Clinical disease was assessed using the well-established EAE scale: (0) no disease; (1) floppy tail; (2) hind limb paresis; (3) hind limb paralysis; (4) quadriplegia; (5) death. Scores that were in-between the clear-cut gradations were scored in increments of 0.5.

### Drug administration and grouping

MP4-immunized mice were treated by intraperitoneal injections of 150 μg anti-CEACAM1 mAb CC1 (mouse IgG1; produced and purified by inVivo BioTech Services) or isotype control mIgG1 (inVivo BioTech Services) every third day for 27.6 ± 5.4 days after disease onset. The grouping of all animals into the respective two groups was done randomly. A total of *n* = 8 mice was treated with mCC1, while *n* = 7 mice received mIgG1 in three independent experiments.

### Transcardial perfusion and tissue preparation

mCC1- (*n* = 8) or isotype control- (mIgG1; *n* = 7) treated MP4-immunized mice were sacrificed with CO_2_ on day 27.6 ± 5.4 after disease onset. After specimen collection for ELISPOT and ELISA all mice were perfused transcardially with 4% paraformaldehyde (PFA) (AppliChem GmbH) in 0.1 M phosphate-buffered saline (PBS), pH 7.4. Draining inguinal lymph nodes and cerebellum were then post-fixed at 4 °C for 24 h. All tissues were embedded in paraffin (Leica TP1020) and stored at 4 °C until analysis.

### Paraffin sections

For immunohistochemical stainings paraffin-embedded draining inguinal lymph nodes and cerebella were sectioned serially and transversally on a microtome (Leica SM 2010 R). The cerebella were cut into 100 serial sections at 5 μm, while the lymph nodes were processed into 52 serial sections at 4 μm. The sections were transferred onto SuperFrost Plus microscope slides (Thermo Scientific). The first, middle and last slide with the corresponding sections from each organ was stained with hematoxylin and eosin (H.E.).

### Immunohistochemistry (IHC)

Cerebellar sections that were adjacent to the ones used for H.E. staining were immunohistochemically stained for the B cell surface marker B220 (CD45R) and for CEACAM1. Sections were dewaxed and rehydrated. For acidic demasking of the antigens sections were briefly boiled in citrate buffer (pH 6.0) seven times and subsequently slowly cooled down for 30 min. Because the host species of the primary antibody against murine CEACAM1 (mCC1) was mouse, Vector M.O.M. kit (Vector Laboratories) was used to reduce the background staining. For mCC1 (diluted 1:100) the instructions of the M.O.M. kit were followed and Neutravidin-Dylight550 was used as a fluorescent dye (Thermo Scientific). Afterwards, slides were blocked with 5% normal goat serum (NGS) (Sigma) in PBS. Rat anti-mouse B220 (eBioscience) was diluted 1:300 in PBS and goat anti-rat Cy5 (diluted 1:400 in PBS; Jackson ImmunoResearch Laboratories) was used as a secondary antibody. Slides were covered with n-propyl gallate (Sigma). The morphology of the inguinal lymph nodes was studied by IHC for B cells (rat anti-mouse B220; 1:500; eBioscience), T cells (rabbit anti-mouse CD3; 1:500; abcam), FDCs (rabbit anti-mouse CD35; 1:500; Bioss), high endothelial venules (rat anti-mouse PNAd; 1:200; BD Pharmingen), proliferating cells (rabbit anti-mouse Ki67; 1:500; abcam) and CXCL13 (rabbit anti-mouse CXCL13; 1:80; R&D Systems). To block endogenous peroxidase sections were incubated with 3% hydrogen peroxide at room temperature for 10 min. All sections were blocked with 5% NGS/PBS. The primary antibodies were diluted in PBS (pH 7.4). The appropriate secondary antibodies were biotin-conjugated. All slides were developed with DAB except for the B220 staining, which was detected using Vector Blue substrate. Nuclei of all stainings were stained with nuclear fast red (Sigma). The immunfluorescent staining was observed with a Biorevo BZ-9000 microscope (Keyence). Light microscopic analysis was performed on a Leica DM2000 LED microscope (Leica Microsystems CMS GmbH).

### Quantification of CNS infiltrates

To assess the number of infiltrates in the cerebellar parenchyma 12 sections covering equidistant planes of the entire cerebellum of each mouse were observed using a 5x objective and the parenchymal area was measured with ImageJ 1.48 k (NIH). To determine the amount of different kinds of immune cell infiltrates the sections adjacent to the ones that were stained with H.E. were stained for B220. All visible infiltrates were observed with a 10x objective. The amount of B cell aggregates, diffuse B cell infiltrates and non-B cell infiltrates was subsequently counted and the area of the B cell aggregates was measured with ImageJ 1.48 k (NIH).

### B cell enzyme-linked immunospot assay (ELISPOT)

Inguinal draining lymph nodes of mCC1- (*n* = 8) or mIgG1- (*n* = 7) treated MP4-immunized mice were collected on day 27.6 ± 5.4 after disease onset, disintegrated mechanically and filtered through a 70 μm Falcon cell strainer (BD Biosciences). Cells were washed twice with RPMI-1640 (Biochrom AG) and resuspended in HL-1 (Lonza) containing 1% L-glutamine (Sigma) and 1% penicillin/streptomycin (Sigma). MultiScreen^®^HTS 96-well ELISPOT plates (Merck Millipore) were coated in duplicate wells with either 10 μg/ml MP4 or 15 μg/ml anti-mouse IgG (MabTech). PBS alone (Biochrom) served as negative control coating. Plates were blocked with 10% fetal bovine serum (FBS; Gibco) in sterile PBS at room temperature for 2 h. Cells were plated at 1 × 10^6^ cells/well and incubated at 37 °C and 7% CO_2_ for 24 h. Biotin-conjugated goat anti-mouse IgG (Dako) was used as secondary antibody at 1:2,000 dilution in 0.5% FBS/PBS. After incubation with Streptavidin-AP (Vector Laboratories) at 1:800 dilution in 0.5% FBS/PBS for 2 h, plates were developed with Vector Blue substrate (Vector Laboratories). The spots were counted on an ImmunoSpot Series 6 UV Analyzer (CTL-Europe).

### Enzyme-linked immunosorbent assay (ELISA)

Blood samples of the mCC1- (*n* = 8) or mIgG1-treated (*n* = 7) MP4-immunized mice were obtained on day 27.6 ± 5.4 after disease onset and stored at 4 °C for two days to achieve full blood coagulation without degradation of serum proteins. The serum was separated from the cellular components by centrifugation (15,000 g, 15 min) and stored at –80 °C. MaxiSorp plates (Nunc Thermo Scientific) were coated with 10 μg/ml MP4 at 4 °C overnight. Plates were blocked with 1% milk powder (Heirler Cenovis GmbH) diluted in 0.05% Tween (AppliChem GmbH) in PBS at room temperature for 2 h. Serum samples were plated at 1:1,000 dilution in blocking buffer in triplicates and incubated at 4 °C overnight. Blocking buffer alone served as a negative control. Biotin-conjugated goat anti-mouse IgG (eBioscience) was used as secondary antibody at 0.625 μg/ml in 0.1% milk powder/PBS/0.05% Tween and incubated at 4 °C overnight. After incubation with Streptavidin-HRP (BD Pharmingen; 1:1,000 in 0.1% milk powder/PBS/0.05% Tween) for 2 h the plates were developed with TMB substrate solution (eBioscience). The reaction was stopped with 1% sulphoric acid. The optical density was measured at 450 nm on a Victor 3 multilabel counter (PerkinElmer).

### Murine *in vitro* B cell aggregation assays

For murine B cell enrichment the spleens of *n* = 3 wild-type B6 mice per individual experiment were disintegrated mechanically and filtered through a 70 μm Falcon cell strainer (BD Biosciences). Cells were washed twice with RPMI-1640 (Biochrom). To enrich B cells a mouse B lymphocyte enrichment set (BD IMag^TM^) for negative selection was used. The enriched B cells were plated at a concentration of 5 × 10^5^ cells per well into 96-well cell culture plates. Cells were preincubated with mCC1 antibody at 200 μg/ml for 90 min prior to a three-day incubation period with 25 μg/ml LPS + 5 U/ml IL-4. mIgG1 was used as an isotype control antibody for mCC1. The remaining amount of single cells was counted using the ITCN plugin for ImageJ 1.48 k (NIH) software analyzing at least 10 individual images per culture condition and experiment, which were acquired on a Leica DMIL LED (Leica Microsystems) microscope.

### Patients

MS diagnosis was established according to the 2005 McDonald criteria. All patients additionally fulfilled the 2010 revised criteria^29^. Patients with a history of other autoimmune diseases and severe accompanying systemic disorders were excluded from the study. Likewise, patients that had undergone plasmapheresis, B cell depletion therapy, intravenous immunoglobulin or immunosuppressive treatment 12 months prior to the inclusion into the study were also excluded. All patients in remission were on natalizumab treatment at the time of the blood draw. Natalizumab is a monoclonal anti-VLA4 antibody that blocks the migration of lymphocytes through the blood-brain-barrier. Since untreated MS patients were unavailable to us we favored treatment with natalizumab over the other available MS drugs, which are primarily immunomodulatory in their nature, while natalizumab is not. All experiments on human material were approved by the corresponding ethics review committee for the Caritas-Krankenhaus Bad Mergentheim, the Klinikum Augsburg and the University Hospitals of Würzburg (Bayerische Landesärztekammer: approval number mb BO 14043 and ethics review committee of the University of Würzburg: approval number 258/14). Written informed consent was obtained from each patient and healthy donor. The methods were carried out in accordance with the approved guidelines.

### CEACAM1 staining of B and T cell subsets by flow cytometry

PBMCs were isolated by density gradient centrifugation from *n* = 19 healthy controls, *n* = 19 RRMS patients in remission that were treated with natalizumab and *n* = 8 RRMS patients during relapse, of which *n* = 5 patients were untreated, *n* = 1 patient received natalizumab and *n* = 2 patients interferon-β. Additionally, PBMCs from *n* = 15 healthy controls and from *n* = 15 (B cell group) or *n* = 22 (T cell group) RRMS patients, respectively, were tested after B cell stimulation to investigate CEACAM1 expression on lymphocyte subsets. Furthermore, *n* = 8 healthy controls as well as *n* = 7 (B cell subset group) and *n* = 10 (T cell subset group) RRMS patients were analyzed after B cell stimulation. PBMCs were cultured in RPMI-1640 supplemented with 1% L-glutamine (Sigma-Aldrich), 1% penicillin/streptomycin (Sigma-Aldrich), 10% FBS (Biochrom AG), IL-2 at 15 ng/ml (Peprotech), R-848 at 2.5 μg/ml (Enzo Life Sciences) and 1 μmol/l β-mercaptoethanol (Sigma-Aldrich) for 96 h at 37 °C and 7% CO_2_, according to the protocol described before^30^. Detailed characteristics of all healthy controls and RRMS patients are listed in Supplementary Tables 1–3. All samples were stained and analyzed immediately or after stimulation, respectively. The following anti-human antibodies were used (all from BD Biosciences): anti-CD3 (APC-H7, clone SK7), anti-CD4 (PE-Cy™7, clone SK3), anti-CD8 (BD-Horizon™, V500, clone SK1), anti-CD20 (PerCP-Cy™5.5, clone 2H7), anti-CD27 (APC, clone M-T271), anti-CD43 (PE, clone 1G10), IgG1 isotype control conjugated with PE and FITC (clone MOPC-21). Anti-human CD66a/CEACAM1-FITC was from Diaclone (clone B-D60). Cells were also stained with BD Horizon™ Fixability Viability Stain 450 (FVS450) prior to extracellular staining. Stained cells were analyzed on a FACS Canto™ II (BD Biosciences) at a flow rate of 2,000 events per second and each tube was run until 200,000 events were recorded. Data were analyzed using FlowJo version 10.0.6 (Tree Star, Inc.). In order to identify different B cell subpopulations, we used the gating strategy as first published by Griffin *et al*.^31^ with some modifications. We excluded dead cells before a single gate on the FSC-H (forward scatter height)/FSC-A (forward scatter area) profile was set followed by a single cell gate on the SSC-H (sideward scatter height)/SSC-A (sideward scatter area) profile. We then excluded CD3^+^ cells and defined CEACAM1^+^ B cells as CD3^−^CD20^+^CD66a^+^. CEACAM1^+^ B cell subgroups were specified as CEACAM1^+^ memory B cells (CD20^+^CD27^+^CD66^+^), CEACAM1^+^ naïve B cells (CD20^+^CD27^−^CD66^+^) and CEACAM1^+^ B1 cells (CD20^+^CD27^+^CD43^+^CD66^+^). Gates were first set identically for all samples followed by adjustments based on fluorescence minus one controls as previously described^32^ and isotype controls for CD43.

### CEACAM1/TIM3 staining of B and T cell subsets by flow cytometry

PBMCs were isolated by density gradient centrifugation and stained immediately and after T cell stimulation with mouse anti-human anti-CD3 (clone CD3-2; Mabtech) and rat anti-human CD28 (clone CD28-A; Mabtech) at 0.1 μg/ml for 72 h at 37 °C and 7% CO_2._ Cells were from *n* = 8 (unstimulated), or *n* = 7 (stimulated) healthy controls and from *n* = 10 (unstimulated) or *n* = 8 (stimulated) RRMS patients in remission, respectively, that were treated with natalizumab. Detailed characteristics of all RRMS patients and healthy controls are listed in Supplementary Table 4. The following anti-human antibodies were used (all from BD Biosciences): anti-CD3 (APC-H7, clone SK7), anti-CD4 (PE-Cy™7, clone SK3), anti-CD8 (BD-Horizon™, V500, clone SK1), anti-CD20 (PerCP-Cy™5.5, clone 2H7). Anti-TIM-3-APC (clone F38-2E2) was from eBioscience (Affymetrix) and anti-human CD66a/CEACAM1-FITC was from Diaclone (clone B-D60). Cells were also stained with BD Horizon™ Fixability Viability Stain 450 (FVS450) prior to extracellular staining. Stained cells were analyzed on a FACS Canto™ II (BD Biosciences) at a flow rate of 2,000 events per second and each tube was run until a minimum of 50,000 events was recorded. Data were analyzed using FlowJo version 10.0.6 (Tree Star, Inc.). We excluded dead cells before a single gate on the FSC-H (forward scatter height)/FSC-A (forward scatter area) profile was set followed by a single cell gate on the SSC-H (sideward scatter height)/SSC-A (sideward scatter area) profile. We then set a CD3/CD20 dichotomous gate to investigate T cells (CD3^+^) and B cells (CD20^+^). Subsequently, we defined CTLs and T_H_ cells by setting a CD4/CD8 bivariant gate. CEACAM1^+^/TIM-3^+^ CTLs were defined as CD3^+^CD20^−^CD8^+^CD66a^+^TIM-3^+^ and CEACAM1^+^/TIM-3^+^ T_H_ cells were specified as CD3^+^CD20^−^CD4^+^CD66a^+^TIM-3^+^. CEACAM1^+^/TIM-3^+^ B cells were identified as CD3^−^CD20^+^CD66a^+^TIM-3^+^. Gates were first set identically for all samples followed by adjustments based on fluorescence minus one controls as previously described^32^.

### IHC of human brain sections

Seven μm thick paraffin sections of human MS brain (*n* = 12) and control brain tissue (*n* = 12) were obtained from the Multiple Sclerosis and Parkinson’s Tissue Bank, Centre for Brain Sciences, Imperial College London. Paraffin-embedded tissue from normal human prostate was obtained from the Department of Pathology, University Hospital of Hamburg-Eppendorf, Hamburg. The sections were dehydrated in a descending alcohol series prior to IHC. Epitope retrieval was performed in 0.1 M citrate buffer. Sections were blocked with 5% NGS (Vector Laboratories) in PBS at room temperature for 2 h. Sections were then incubated with the primary antibodies directed against CEACAM1 (clone C5-1X; Reliatech; diluted 1:50) and CD20 (Fisher Scientific; diluted 1:400) in PBS at 4 °C overnight. As a control, sections were incubated in the absence of primary antibody. The next day, sections were stained with goat anti-rabbit Cy5 (Dianova; diluted 1:300) and goat anti-mouse Cy3 (Dianova; diluted 1:600) at room temperature for 2 h. Counterstaining of cellular nuclei was performed by incubation with 4′,6-diamidino-2-phenylindole (DAPI, Roche; diluted 1:5,000 in PBS). Sections were analyzed on a Keyence Biorevo BZ-9000 microscope.

### Human *in vitro* B cell aggregation assays

PBMCs were obtained from *n* = 4 MS patients that had been treated with natalizumab for at least six months. In addition, enriched human B cells were collected from *n* = 4 age- and gender-matched and *n* = 9 additional healthy controls. The latter were used for titration experiments and antibody testing. For the enrichment of human B cells RosetteSep cocktail (Stemcell Technologies) for negative selection was used. The enriched B cells were plated at 1.8 × 10^5^/ml into 24-well cell culture plates. After preincubation with anti-CEACAM1 antibody for 90 min aggregation was induced by 16 h of incubation with mouse anti-human CD19 (1 μg/ml; BD Pharmingen). The three clones C5-1X (Reliatech), 4D1/C2 (produced and purified in-house) and T84.1 (inVivo BioTech Services) were tested at different concentrations (150 μg/ml, 250 μg/ml and 400 μg/ml). Mouse anti-human IgG1 (BD Pharmingen) was used as an isotype control antibody for all anti-CEACAM1 antibodies. Thirteen images were acquired from each well using a Leica DMIL LED (Leica Microsystems CMS GmbH) microscope. Aggregation was quantified with ImageJ 1.48 k (NIH).

### Statistics

Except for the analysis of the EAE clinical data the Wilcoxon rank sum test on Prism (Graph Pad Prism4 and Prism6, Graph Pad Software) was used. Statistical differences between EAE scores were calculated using one-way ANOVA on SigmaPlot software version 12.0 (Systat Software, Inc.). Statistical significance was defined as *P* ≤ 0.05. Spearman correlation analyses between two variables were applied and a correlation coefficient (r_s_) of *P* < 0.05 (two-sided tests) was considered as statistically significant.

## Additional Information

**How to cite this article**: Rovituso, D. *et al*. CEACAM1 mediates B cell aggregation in central nervous system autoimmunity. *Sci. Rep*. **6**, 29847; doi: 10.1038/srep29847 (2016).

## Supplementary Material

Supplementary Information
