# Supplementary material for: CEACAM1 mediates B cell aggregation in central nervous system autoimmunity
Source: Sci Rep. 2016 Jul 20;6:29847. doi: 10.1038/srep29847 (PMC4951702; doi:10.1038/srep29847)
Supplement: Supplementary Information [file srep29847-s1.pdf]

CEACAM1 mediates B cell aggregation in central nervous system autoimmunity

Damiano Rovituso[1], Laura Scheffler[1], Marie Wunsch[1], Christoph Kleinschnitz[2], Sebastian Dörck[2], Jochen Ulzheimer[3], Antonios Bayas[4], Lawrence Steinman[5], Süleyman Ergün[1*] & Stefanie Kuerten[1*]

[1]Department of Anatomy and Cell Biology, University of Würzburg, Würzburg, Germany.

[2]Department of Neurology, University Hospitals of Würzburg, Würzburg, Germany.

[3]Department of Neurology, Caritas-Krankenhaus Bad Mergentheim, Bad Mergentheim, Germany.

[4]Department of Neurology, Klinikum Augsburg, Augsburg, Germany.

[5]Department of Neurology, Stanford University School of Medicine, Palo Alto, CA, USA.

D.R. and L.S. contributed equally to this work.

S.E. and S.K. contributed equally to this work.

*Corresponding authors: Stefanie Kuerten, M.D., Ph.D. or Süleyman Ergün, M.D., Department of Anatomy and Cell Biology, Koellikerstraße 6, 97070 Würzburg, Germany. Phone: +49-931-31-85998; Fax: +49-931-31-82087 stefanie.kuerten@uni-wuerzburg.de, sueleyman.erguen@uni-wuerzburg.de.

SUPPLEMENTARY MATERIALS

SUPPLEMENTARY METHODS

Polychromatic flow cytometry of murine B cell activation and inhibition markers. To investigate the effect of the anti-CEACAM1 antibody mCC1 on murine B cell activation and inhibition markers, we stained splenic B cells for flow cytometry after 90 min of incubation with either mCC1 or mIgG1. Pooled spleens of n = 3 wild-type B6 mice in each experiment were disintegrated mechanically and filtered through a 70 µm Falcon cell strainer (BD Biosciences). Cells were washed twice with RPMI-1640 (Biochrom). We enriched murine B cells (BD IMag™) and plated them at 5 x 10^5 cells per well using 96-well cell culture plates. Cells were either incubated with mCC1 antibody or mIgG1 at 200 µg/ml in RPMI-1640 (10 % FCS) at 37°C, 7 % CO_2 for 90 min. Additional controls contained no antibodies and were also incubated for 90 min. Alternatively, cells were stimulated with LPS (25 µg/ml) and IL-4 (5 U/ml) for 72 h prior to or after 90 min of mCC1 *vs.* mIgG1 incubation. The following anti-mouse antibodies were used (all from BD Biosciences): anti-CD22.2 (FITC, Lyb-8.2), anti-B220/CD45R (BV510, RA3-6B2), anti-CD80 (BV421, 16-10A1), anti-CD40 (APC, 3/23), anit-CD86 (PE, GL1). Cells were also stained with BD Horizon™ Fixability Viability Stain 780 (FVS780) prior to extracellular staining. Stained cells were analyzed on a FACS Canto™ II (BD Biosciences) at a flow rate of 100-1,000 events per second and each tube was run until 50,000 events were recorded. Data were analyzed using FlowJo version 10.0.6 (Tree Star, Inc.). To identify differences between marker expression comparing mCC1- and mIgG1-treated cells dead cells were excluded and a gate was subsequently set on the FSC-H/FSC-A and SSC-H/SSC-A profile to gate single cells. We then

set a gate on all B220$^+$ cells. Finally, B220$^+$ cells were analyzed to determine the median fluorescence intensity (mfi) of CD22, CD40, CD80 and CD86 expression.

TIM-3 PCR. PBMCs were isolated from *n* = 6 RRMS patients and *n* = 3 age- and gender-matched healthy controls. For the enrichment of human B cells RosetteSep cocktail (Stemcell Technologies) for negative selection was used. All RRMS patients were treated with natalizumab. Primers for TIM-3 and GAPDH primers were purchased from Eurofins MWG Operon. *hTIM-3*: forward primer, 5´-CAGATACTGGCTAAATGGG-3´, reverse primer, 5´-CTTGGCTGGTTTGATGAC-3´. *hGAPDH*: forward primer, 5´-TGATGACATCAAGAAGGTGG-3´, reverse primer, 5´-TTTCTTACTCCTTGGAGGCC-3´. Cycling conditions were the following: 95 °C × 5 min (1 cycle), 35 cycles of 95 °C, 59 °C and 72 °C × 30 s. 72 °C × 2 min (1 cycle) and 4 °C hold. The TIM-3 fragment amplicon was 166 bp and the GAPDH fragment amplicon 248 bp in length.

Cytospin preparation. Cytospin of 0.25 x 10^6 enriched human B cells from healthy donors were prepared for immunocytochemistry (ICC). For the enrichment of human B cells RosetteSep cocktail (Stemcell Technologies) for negative selection was used. A total of 200 µl cell suspension was loaded on each cuvette and spun at 190 x g for 10 min. Cytospins were fixed in 4 % paraformaldehyde for 10 min and stored at 4 °C overnight. On the next day, cytospins were permeabilized with ice-cold 100 % methanol at 20 °C for 10 min. Every section was washed twice with 0.1 M phosphate-buffered saline (PBS) and incubated with 10 % normal donkey serum (Dianova) in PBS for 1 hour. Every sample was incubated with primary antibodies diluted in 2 % bovine serum albumin (BSA)/PBS (goat polyclonal anti-human TIM-3 at 10 µg/ml and mouse monoclonal anti-human CD20 (clone L26) at 2.5 µg/ml, both obtained from abcam) at 4

°C overnight. Sections were then stained with donkey anti-goat Cy3 (Dianova; diluted 1:600) followed by incubation with goat anti-mouse Cy5 (Dianova; diluted in 2 % PBS 1:400) at room temperature for 1 h. Counterstaining of cellular nuclei was performed by incubation with 4',6-diamidino-2-phenylindole (DAPI; Roche; diluted 1:5,000 in PBS). Sections were analyzed with a Plan Apo TIRF oil 100x objective (NA:1.5) on a Nikon Eclipse TI-E inverse confocal microscope equipped with laser lines: 405, 561, 647. NIS-Elements Advanced Research Software (Nikon) was used for analysis.

Supplementary Table 1. Characteristics of healthy controls and RRMS patients that were recruited for flow cytometric analysis of unstimulated B cells

	**HC** (*n* = 19)	**RRMS Remission** (*n* = 19)	**RRMS Relapse** (*n* = 8)
**Age at time of testing (years mean ± s.d.)**	37.11 ± 11.70	37.47 ± 9.64	28.50 ± 5.24
**Age at onset (years mean ± s.d.)**	N/A	30.63 ± 8.15	23.33 ± 6.05
**Female to male ratio**	2.8	3.75	3
**Female**	14	15	6
**Male**	5	4	2
**EDSS (mean ± s.d.)**	N/A	3.2 ± 1.7	3.4 ± 1.7 (*n* = 7)
**Time since last relapse (months mean ± s.d.)**	N/A	24.84 ± 23.24	N/A
**Treatment duration (months mean ± s.d.)**	N/A	25.21 ± 24.71	16.67 ± 8.96 (*n* = 3)

N/A = Not applicable.

Supplementary Table 2. Characteristics of healthy controls and RRMS patients that were recruited for flow cytometric analysis of stimulated B and T cells

	**HC[a,b]** (*n* = 15)	**RRMS Remission[a]** (*n* = 15)	**RRMS Remission[b]** (*n* = 22)
**Age at time of testing (years mean ± s.d.)**	36.13 ± 10.01	39.60 ± 10.97	40.00 ± 10.80
**Age at onset (years mean ± s.d.)**	N/A	30.47 ± 8.14	31.14 ± 7.88
**Female to male ratio**	4	4	2.15
**Female**	12	12	15
**Male**	3	3	7
**EDSS (mean ± s.d.)**	N/A	3.4 ± 2.2	4.2 ± 4
**Time since last relapse (months mean ± s.d.)**	N/A	29.40 ± 26.66	25.10 ± 24.13
**Treatment duration (months mean ± s.d.)**	N/A	28.67 ± 27.67	25.45 ± 24.20

[a]B cells were analyzed after 72 h of stimulation with R-848 and IL-2.

[b]T cells were analyzed after 72 h of stimulation with R-848 and IL-2.

N/A = Not applicable.

Supplementary Table 3. Characteristics of healthy controls and RRMS patients that were recruited for flow cytometric analysis of stimulated B and T cell subsets

	HC[a,b] (*n* = 8)	RRMS Remission[a] (*n* = 7)	RRMS Remission[b] (*n* = 10)
**Age at time of testing (years mean ± s.d.)**	36.38 ± 12.75	38.86 ± 9.93	39.5 ± 9.54
**Age at onset (years mean ± s.d.)**	N/A	29.43 ± 7.28	30.90 ± 7.1
**Female to male ratio**	7	N/A	4
**Female**	7	7	8
**Male**	1	0	2
**EDSS (mean ± s.d.)**	N/A	2.9 ± 2.2	3.1 ± 1.9
**Time since last relapse (months mean ± s.d.)**	N/A	27.29 ± 28.22	23.90 ± 24.68
**Treatment duration (months mean ± s.d.)**	N/A	26.14 ± 27.43	26.2 ± 23.34

[a]B cell subsets were analyzed after 72 h of stimulation with R-848 and IL-2.

[b]T cell subsets were analyzed after 72 h of stimulation with R-848 and IL-2.

N/A = Not applicable.

Supplementary Table 4. Characteristics of healthy controls and RRMS patients that were recruited for flow cytometric analysis of unstimulated and anti-CD3/anti-CD28 stimulated PBMCs

	**HC[a]** (***n*** **= 8)**	**HC[b]** (***n*** **= 7)**	**RRMS Remission[a]** (***n*** **= 10)**	**RRMS Remission[b]** (***n*** **= 8)**
**Age at time of testing (years mean ± s.d.)**	35.00 ± 10.99	36.00 ± 11.47	38.30 ± 12.56	37.25 ± 10.44
**Age at onset (years mean ± s.d.)**	NA		29.10 ± 8.52	28.00 ± 7.78
**Female to male ratio**	1	1	4	3
**Female**	8	7	8	6
**Male**	0	0	2	2
**EDSS (mean ± s.d.)**	N/A	N/A	2.5 ± 1.7	2.4 ± 1.8
**Time since last relapse (months mean ± s.d.)**	N/A	N/A	22.60 ± 17.58	25.13 ± 18.68
**Treatment duration (months mean ± s.d.)**	N/A	N/A	23.00 ± 17.18	25.25 ± 18.96 (*n* = 4)

[a]PBMCs were unstimulated.

[b]PBMCs were analyzed after 72 h of anti-CD3/anti-CD28 stimulation.

N/A = Not applicable.

Supplementary Table 5. Characteristics of MS patients that were studied by immunohistochemistry for CEACAM1 expression on brain infiltrating B cells

Donor no.	Died at age (years)	Gender (f/m)	Cause of death	Disease duration (years)	Treatment	Diagnosis
**1**	51	M	bronchopneumonia	2	unknown	PPMS
**2**	35	F	MS	5	unknown	SPMS
**3**	46	M	MS, bronchopneumonia	20	unknown	SPMS
**4**	44	F	septicaemia, pneumonia	19	no treatment	SPMS
**5**	39	M	pneumonia, sepsis	10	IFN-β	SPMS
**6**	53	F	MS	18	IFN-β	unknown
**7**	49	M	renal failure	20	unknown	SPMS
**8**	39	F	bronchopneumonia, MS	13	no treatment	PPMS
**9**	57	F	bronchopneumonia, advanced MS	29	unknown	PPMS
**10**	38	F	pneumonia, MS	22	azathioprine	SPMS
**11**	63	F	bronchopneumonia, MS	32	unknown	SPMS
**12**	45	F	MS	25	alemtuzumab	SPMS

Supplementary Table 6. Characteristics of healthy controls and RRMS patients that were recruited for B cell aggregation assays

	**HC[a] (*n* = 4)**	**HC[b] (*n* = 3)**	**HC[c] (*n* = 9)**	**HC[d] (*n* = 4)**	**RRMS (*n* = 4)**
**Age at time of testing (years mean ± s.d.)**	26.50 ± 3.51	25.67 ± 3.79	35.56 ± 15.31	44.50 ± 16.78	41.00 ± 18.26
**Age at onset (years mean ± s.d.)**	N/A	N/A	N/A	N/A	29.50 ± 11.50
**Female to male ratio**	1.0	0.5	3.0	1.0	1.0
**Female**	2.0	1.0	6.0	2.0	2.0
**Male**	2.0	2.0	2.0	2.0	2.0
**EDSS (mean ± s.d.)**	N/A	N/A	N/A	N/A	3.63 ± 2.56
**Time since last relapse (months mean ± s.d.)**	N/A	N/A	N/A	N/A	19.00 ± 11.63
**Treatment duration (months mean ± s.d.)**	N/A	N/A	N/A	N/A	18.00 ± 11.52

[a]Healthy controls used for testing of the anti-CEACAM1 clone C5-1X.

[b]Healthy controls used for testing of the anti-CEACAM1 clone 4D1/C2.

[c]Healthy controls used for testing of the anti-CEACAM1 clone T84.1.

[d]Age- and gender-matched healthy controls.

N/A = Not applicable.

SUPPLEMENTARY FIGURES

Supplementary Figure 1

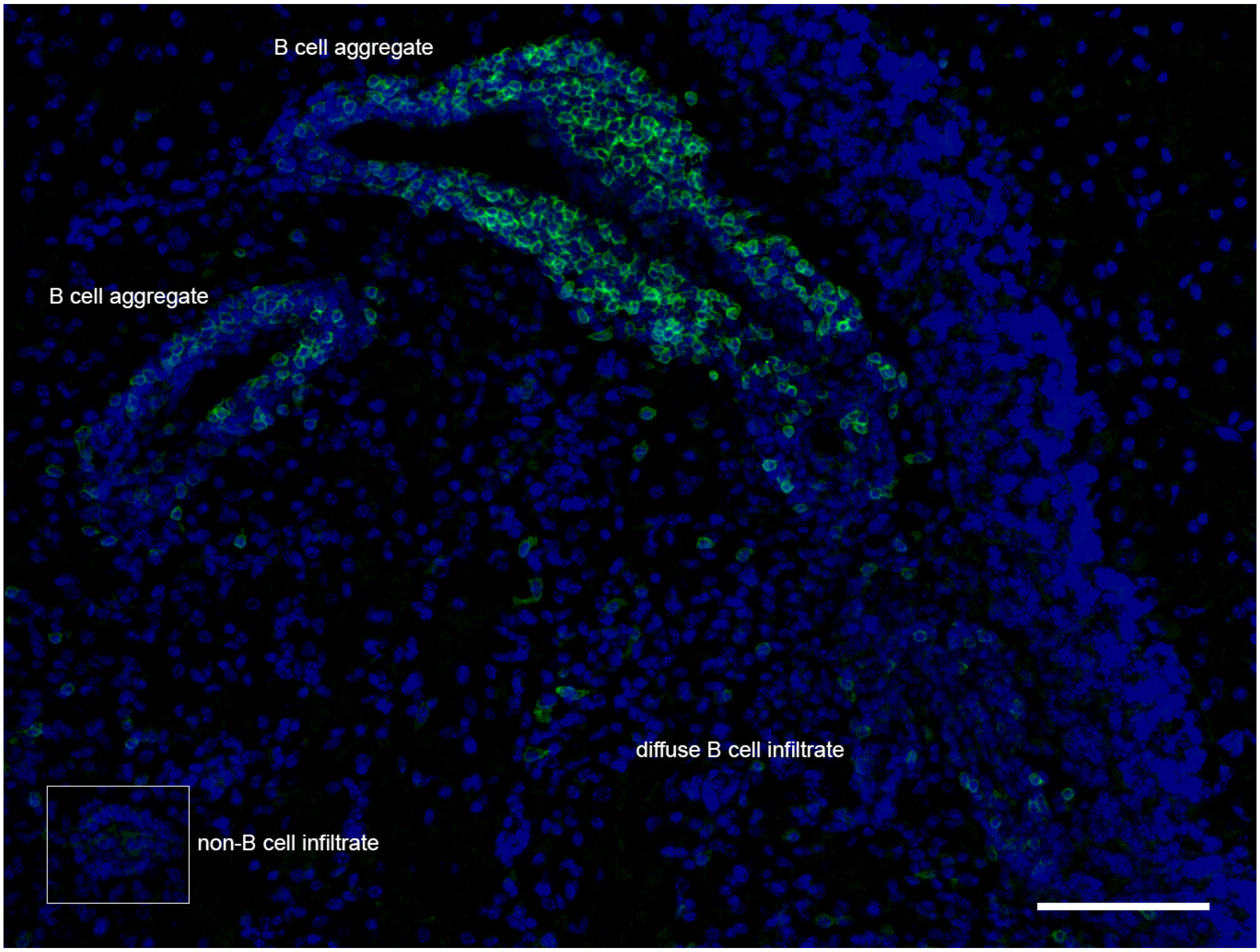

Supplementary Figure 1. B cell infiltrate classification in MP4-induced EAE. B6 mice were immunized with MP4 and cerebellar sections were obtained 30 days after disease onset. Scale bars: 50 µm.

Supplementary Figure 2

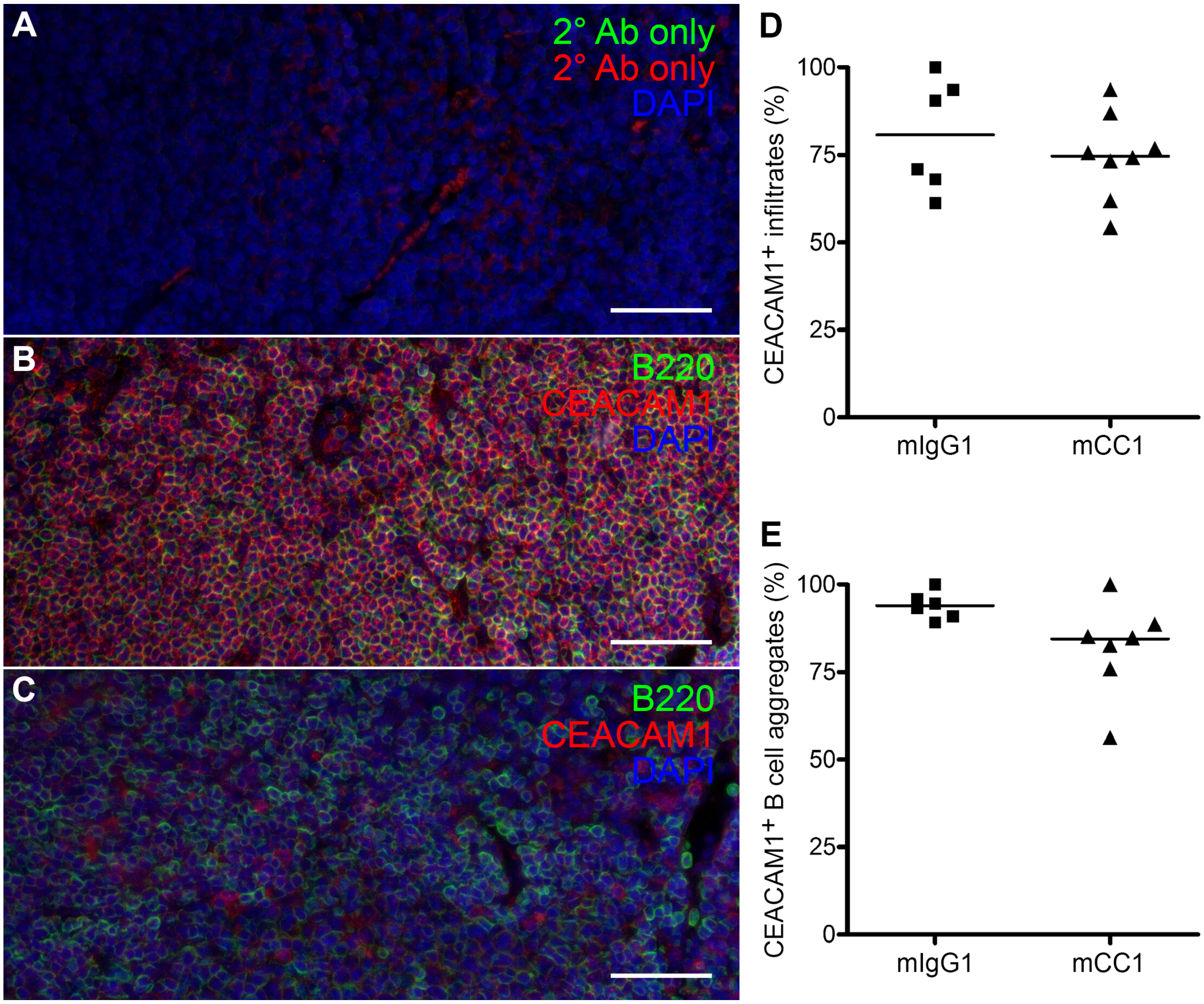

Supplementary Figure 2. CEACAM1 expression on B cells after treatment with mCC1 *vs.* mIgG1 isotype control. (**A-C**) Representative IHC staining of lymph node tissue from MP4-immunized mice after 30 days of treatment with mCC1 *vs.* mIgG1. Scale bars: 50 µm. (**D**) Percentage of CEACAM1^{+} cerebellar infiltrates after treatment with mCC1 *vs.* mIgG1. (**E**) Percentage of CEACAM1^{+} cerebellar B cell aggregates after treatment with mCC1 *vs.* mIgG1.

Supplementary Figure 3

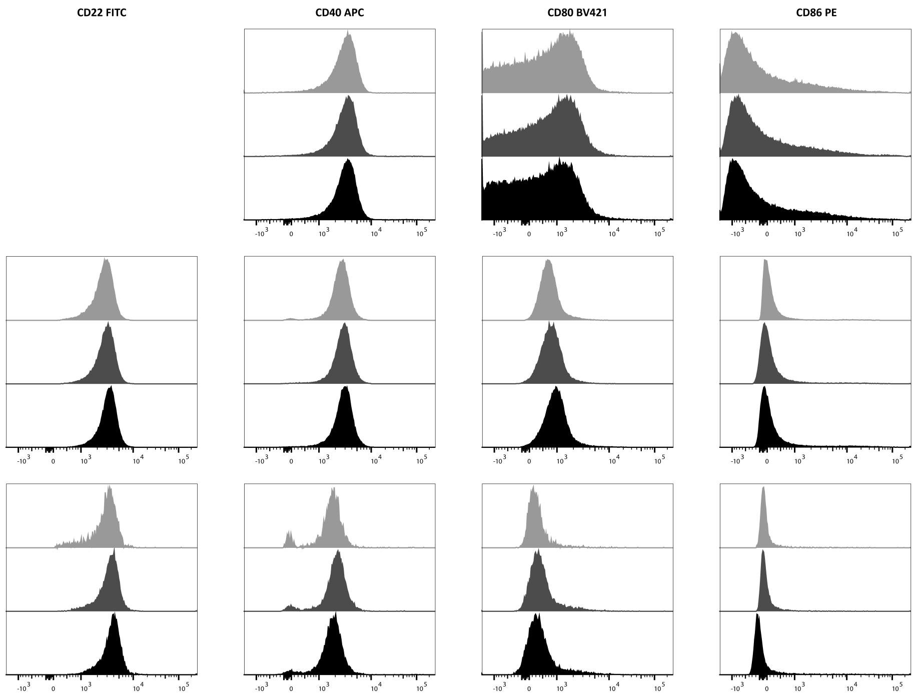

	Sample Name	Subset Name	MFI CD40 APC	MFI CD80 BV421	MFI CD86 PE
	control	B cells	3052	631	-389
	isotype control	B cells	3132	779	-331
	mCC1	B cells	3113	377	-407

	Sample Name	Subset Name	MFI CD22 FITC	MFI CD40 APC	MFI CD80 BV421	MFI CD86 PE
	control	B cells	2702	2563	713	158
	isotype control	B cells	2911	2804	803	133
	mCC1	B cells	3246	2888	980	143

	Sample Name	Subset Name	MFI CD22 FITC	MFI CD40 APC	MFI CD80 BV421	MFI CD86 PE
	control	B cells	3095	1784	294	46,2
	isotype control	B cells	3490	2140	414	56,5
	mCC1	B cells	3823	1825	374	-87,4

Supplementary Figure 3. Effects of mCC1 on murine B cell activation. Enriched splenic murine B cells were incubated with 200 µg/ml mCC1, mIgG1 isotype control or without antibody for 90 min. Each experiment was performed with pooled cells from *n* = 3 mice. The median fluorescence intensity (mfi) is given for each B cell surface marker: anti-CD22 FITC, anti-CD40 APC, anti-CD80 BV421 and anti-CD86 PE.

Supplementary Figure 4

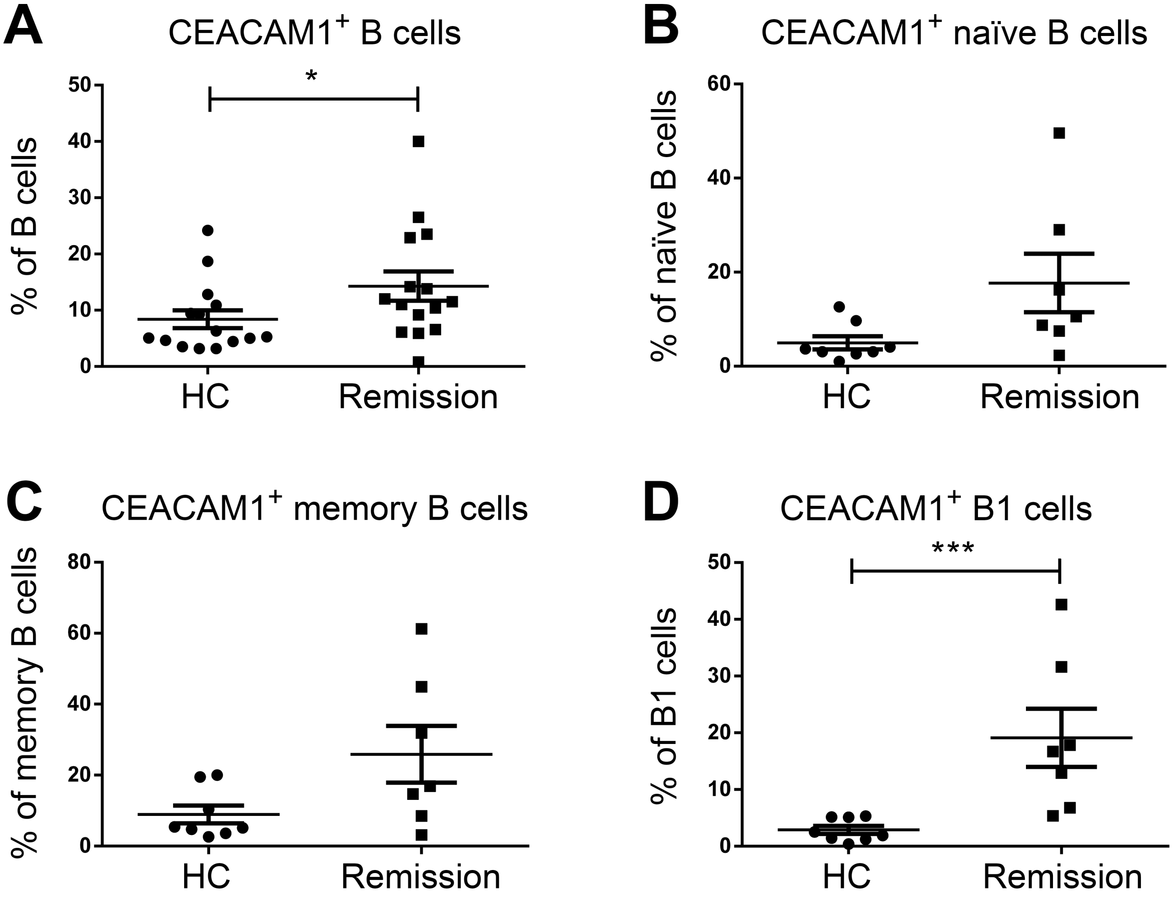

Supplementary Figure 4. Flow cytometric analysis of CEACAM1 expression on peripheral B cells from healthy controls and RRMS patients after polyclonal stimulation. (**A**) CEACAM1 expression on B cells, naïve (**B**) and memory B cells (**C**) as well as on B1 cells (**D**). MS patients were tested during remission (**A**, n = 15; **B** to **D**, n = 7). All patients in remission received treatment with natalizumab. Healthy controls (**A**, n = 15; **B** to **D**, n = 8) were age- and gender-matched. Statistical significance was determined by Mann-Whitney test. Detailed information on patient and healthy control characteristics are provided in Supplementary Tables 2 and 3.

Supplementary Figure 5

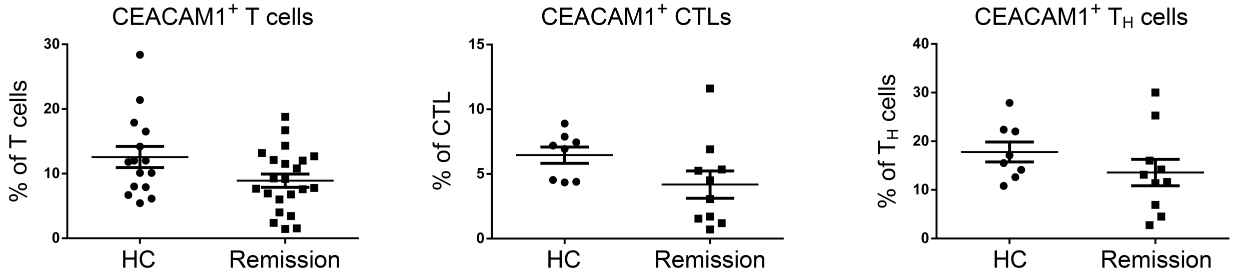

Supplementary Figure 5. Flow cytometric analysis of CEACAM1 expression on peripheral T cells from healthy controls and RRMS patients. (**A**) CEACAM1 expression on T cells, cytotoxic T lymphocytes (**B**, CTLs) and T helper cells (**C**, T_H). RRMS patients were tested during remission (**A**, n = 22; **B** to **C**, n = 10). Healthy controls (**A**, n = 15; **B** to **C**, n = 8) were age- and gender-matched. Statistical significance was determined by Mann-Whitney test. Detailed information on healthy control and patient characteristics are provided in Supplementary Tables 2 and 3.

Supplementary Figure 6

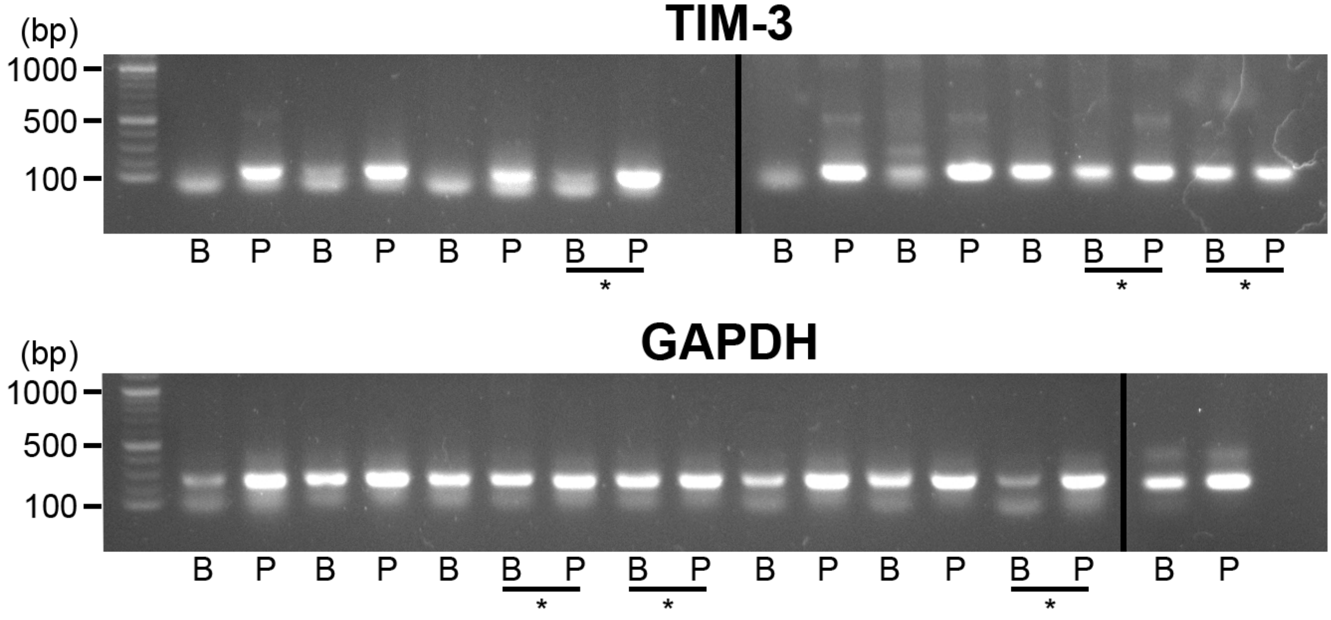

Supplementary Figure 6. TIM-3 expression in human B cells. PCR results showing TIM-3 (upper row) and GAPDH (bottom) expression in RRMS patients and healthy controls (*****). The panels display the expression of TIM-3 or GAPDH from enriched human B cells (**B**) or PBMCs (**P**), respectively, each from the same individual. TIM-3 PCR amplicon size was 166 bp and 248 bp for GAPDH. The lanes were run on the same gel but were noncontiguous.

Supplementary Figure 7

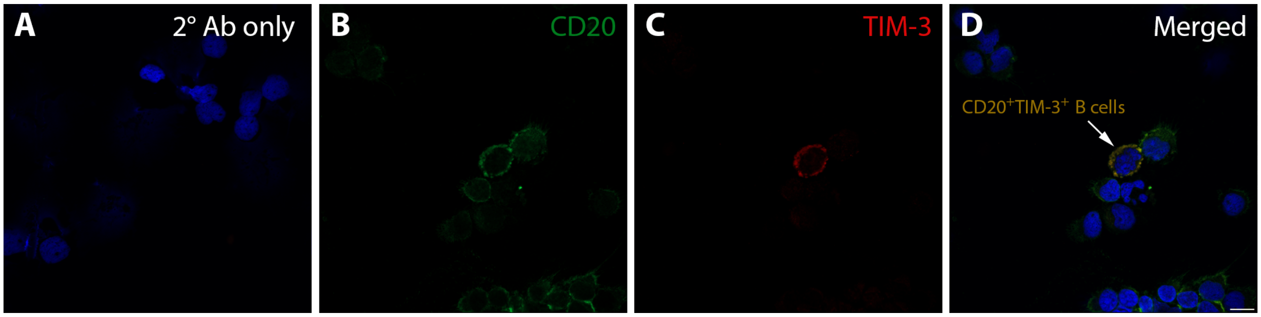

Supplementary Figure 7. CD20 and TIM-3 co-expression on human B cells in a healthy donor. Enriched B cells were stained with mouse anti-human CD20 (clone L26) and goat anti-human TIM-3 (polyclonal). (**A**) Negative controls were incubated with secondary antibody only. Confocal microscopy of $CD20^+$ (**B**) and $TIM\text{-}3^+$ (**C**) cells. (**D**) Merged image. White arrows indicate $CD20^+TIM\text{-}3^+$ B cells.
